# Deep biomarkers of aging and longevity: from research to applications

**DOI:** 10.18632/aging.102475

**Published:** 2019-11-25

**Authors:** Alex Zhavoronkov, Ricky Li, Candice Ma, Polina Mamoshina

**Affiliations:** 1 Insilico Medicine, Hong Kong Science and Technology Park, Hong Kong, China; 2 The Buck Institute for Research on Aging, Novato, CA 84945, USA; 3 The Biogerontology Research Foundation, London, UK; 4 Sinovation Ventures, Beijing, China; 5 Sinovation AI Institute, Beijing, China; 6 Deep Longevity, Ltd, Hong Kong Science and Technology Park, Hong Kong, China

**Keywords:** artificial intelligence, aging clock, deep learning, deep aging clocks, aging biomarkers

## Abstract

Multiple recent advances in machine learning enabled computer systems to exceed human performance in many tasks including voice, text, and speech recognition and complex strategy games. Aging is a complex multifactorial process driven by and resulting in the many minute changes transpiring at every level of the human organism. Deep learning systems trained on the many measurable features changing in time can generalize and learn the many biological processes on the population and individual levels. The deep age predictors can help advance aging research by establishing causal relationships in non-linear systems. Deep aging clocks can be used for identification of novel therapeutic targets, evaluating the efficacy of the various interventions, data quality control, data economics, prediction of health trajectories, mortality, and many other applications. Here we present the current state of development of the deep aging clocks in the context of the pharmaceutical research and development and clinical applications.

## INTRODUCTION

The recent hype cycle in artificial intelligence (AI) resulted in substantial investment in machine learning and increase in available talent in almost every industry and country. This wave of increased attention to AI was fueled by the many credible advances in deep learning that allowed machines to outperform humans in multiple tasks, including image and text recognition and as well as in the strategy board game, of Go. The advantage of deep learning (DL) systems is in their ability to learn and generalize from a large number of examples [1]. DL methods rapidly propagated into the many biomedical applications, starting primarily with the imaging, text, and genomic data [2, 3]. The availability of large volumes of data and new algorithms made it possible to use deep learning to start making predictions about the activity and pharmacological properties of small molecules [4], identify mimetics of the known geroprotectors [5, 6], and discover new ones [7]. The new techniques in deep learning converging with the advances in chemoinformatics enable the creation of completely novel molecular structures with the desired properties for the protein targets of interest in record time [8–12]. Many efforts are underway to apply deep learning techniques to predict the outcomes of clinical trials [13, 14]. However slowly, the artificial intelligence technologies started propagating into aging and longevity research and are rapidly increasing in popularity resulting in the formation of dedicated conference sessions and entire conferences [15] and focused reviews [16].

**Figure 1:**
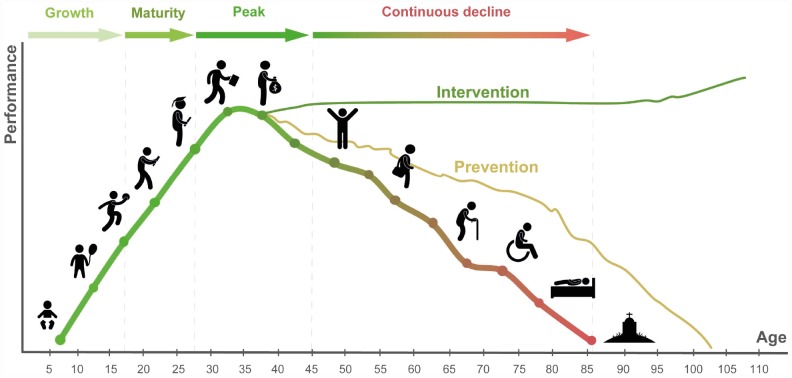
**The general course of human life in the health and performance context.** Preventative strategies may increase lifespan and healthspan. Potential restorative interventions reversing the many biological clocks back to the young productive healthy state may help prevent loss of function and possibly result in future performance gains.

Over many generations humans have evolved to develop from a single-cell embryo within a female organism, come out, grow with the help of other humans, reach reproductive age, reproduce, take care of the young, and gradually decline. Due to the relatively short lifespans early in the evolutionary process, the natural age of peak and optimal performance closely follows puberty and lasts approximately one generation. Considering the average age of the Olympic athlete, the age of optimal performance can be safely defined as 20–30. Human aging is a complex multifactorial process associated with and leading to the gradual decline in all body functions, productivity, psychological changes, multiple diseases and inevitably ending in death (Figure 1).

Lifestyle and behavioral modifications may help slow down the decline and keep the organism in the best possible state for its chronological age, a term commonly referred to as “healthy aging”. To understand the differences between the “healthy aging” and “unhealthy aging”, evaluate the effects of the many lifestyle choices and a variety of emerging longevity interventions, it is essential to be able to track the rate of aging and develop a comprehensive set of aging biomarkers.

### The advent of the aging clocks

There are many biological features that demonstrated correlation with the chronological age such as telomere length [17, 18], racemization of amino acids in proteins [19], and others. The epigenetic age predictors were proposed in 2011 [20]. But it was not until 2012 when the first epigenetic aging clock was published by Hannum [21]. Hannum group used profiled the methylomes derived from peripheral blood samples of healthy individuals to develop the first epigenetic clock consisting of 71 CpG sites and demonstrated the root mean squared error of 4.9 years on independent data. A more precise and comprehensive multi-tissue aging clock was then published in 2013 by Steven Horvath [22] who coined the terms “DNAm clock” and “epigenetic aging clock” and rapidly gained popularity in the aging research community. Horvath used 353 CpG sites and achieved a median error of 3.6 years on the testing set. These clocks were developed using traditional machine learning approaches—notably linear regression with regularization and the use of a limited number of samples. Similar methylation aging clocks were developed for mice [23, 24].

While the epigenetic aging clocks demonstrate spectacular performance in predicting the chronological age, the epigenetic data is not as actionable for target identification or geroprotector discovery. Another abundant and actionable data type is gene expression data. The first transcriptomic aging clock developed on blood-based transcriptomic data was published by Peters et al. in 2015 [25] and extended to other tissues by Yang et al. [26].

In addition to predicting the chronological age and establish the biological relevance of the predictor, it is possible to introduce additional metrics of health status and use the dependent variable. Levine et al. introduced a notion of phenotypic are derived from clinical data [27].

### Deep aging and longevity clocks

Age is a universal feature present in all biological and non-biological material objects and is one of the most abundant features present in almost every data set. While the more traditional machine learning methods were employed to develop aging clocks on multiple data types where the many markers are statistically correlated with chronological age, the advent of deep learning lead to the emergence of next-generation of deep aging and longevity clocks. The deep learning models trained on the large numbers of examples manage to capture the highly non-linear relationships between the seemingly unrelated features.

In addition, the realization that age is a universal feature present in all biological and non-biological objects triggered the interest of the artificial intelligence researchers interested in the study of causality and making the deep neural networks (DNNs) more interpretable. This leads to the convergence of aging research and deep learning [3, 16, 28, 29].

**Figure 2:**
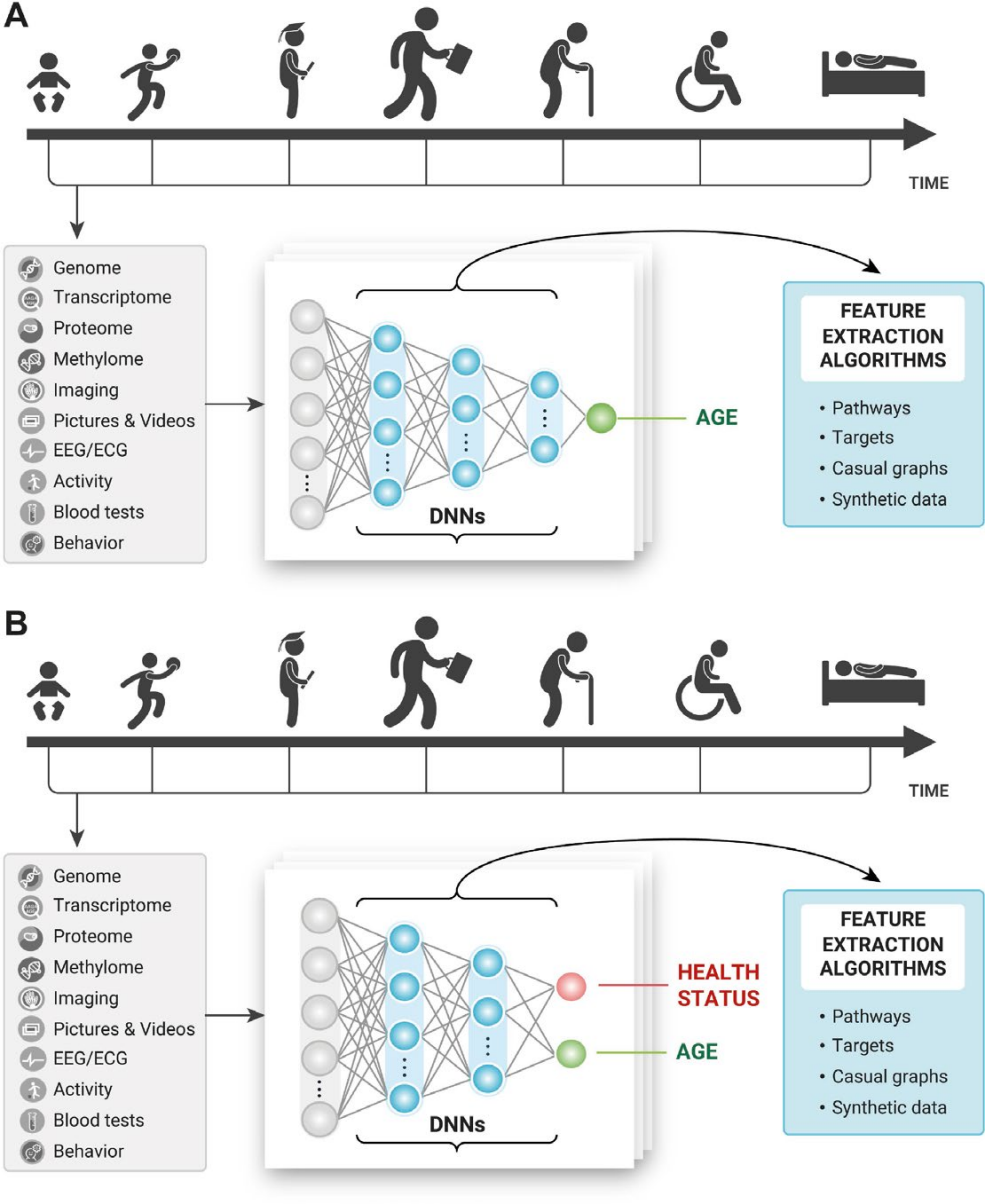
Training the deep neural networks on multimodal longitudinal data to predict (**A**) age of the individual and (**B**) age and health status of the individual and using the feature importance and selection approaches to infer causal relationships, pathways, and targets.

Many of the early works in deep learning for aging research stemmed from the simple thought experiments in how humans perceive age with the various sensory organs. A human can guess with reasonable accuracy the age of another human, other species, material and non-material objects using low- and high-resolution imaging data, movement patterns, even scent and touch. When these data types can be featured and used for training and test in abundant quantity, the deep neural networks should be able to learn the features contributing to age prediction and outperform human accuracy. Most humans can describe the most important features they need to predict someone’s age. For example, the number of wrinkles, grey hair, color of the teeth and many others. The deep neural networks in theory should be able to do it even better and may be used to identify the most important features and reconstruct biological pathways implicated in aging (Figure 2A).

This same thought experiment can be extended to disease-specific biomarkers and disease-specific target identification. A human can quickly recognize that the other person is not feeling well just by looking at the person. A trained physician can make educated guesses about the person’s health status by examining the visual appearance. Many genetic diseases manifest in the very obvious phenotypes and can already be identified using simple imaging data using the deep neural networks [30]. Training to predict age and disease at the same time (Figure 2B) may not only enable more accurate diagnosis and training on fewer disease samples but it may be possible to compare the features and pathways between the age groups, disease stages and predictors trained to predict only age.

These though experiments help generate new hypotheses in inferring causality, multi-target processes and understand the progression of diseases better. This approach also helps combine the multiple data types, understand the relationships between the data types and control the quality of the data [31] and possibly assess its biological relevance [32]. Moreover, it may be possible to use this approach to develop “disease clocks” and track the changing importance in the molecular targets in the context of age and disease. For example, the many diseases (e.g. scleroderma) may start as an autoimmune reaction and then progress into sclerosis, fibrosis and other directions and the importance of the addressable targets may change. The techniques developed to identify the most important features and establish causality using age predictors can be used to identify the most important targets in a specific stage of the disease and personalize interventions.

With the first DNN-based aging clocks published in 2016 by Zhavoronkov laboratory [33], significant progress has been made the past few years in deep learned biomarkers of human aging [28, 29]. The first DL clock was constructed using 41 blood test values of over 50,000 individuals. Making use of DNN abilities to capture nonlinear dependencies between input data and target variable, the initially proposed method was able to achieve mean absolute accuracy of 5.5 years on previously unseen 12,000 individuals. Additionally, this study demonstrated how the deep clock can be used for further interpretations of relations between aging and blood parameters. By employing feature importance analysis they identified top parameters related to age changes.

Later, Mamoshina el al. continued the work and validated the approach on several million anonymized subject records of healthy individuals from three populations: South Korean, Canadian, and Eastern European [34]. The analysis showed that DNNs trained to predict age either on Canadian or Eastern European sample sets predict South Korean samples younger than they chronologically are. Further, for each sample with information on mortality status, the authors observed that subject with slowed aging or predicted younger have a higher life expectancy. Equally, subjects predicted older hence with accelerated aging have a lower life expectancy.

Further validation showed that deep hematological clocks can also be used to evaluate the impact of lifestyle choices on aging. In this manner, Mamoshina et al. demonstrated that tobacco smokers are predicted older than they chronologically are with individuals chronologically younger 55 years predicted twice as old [35]. In addition to age prediction, the authors showed the DNNs can also predict smoking status potentially replacing the error-prone self-reporting.

The rapid development of high-throughput methods enables the advances of DL omics-based aging clocks. While transcriptional data is among the most abundant data types, a large scale transcriptomic analysis remains a challenge [36]. Nevertheless, early in 2018, the first deep transcriptomic aging clock was presented for gene expression profiles of skeletal muscle healthy individuals [37]. The best model achieved mean absolute error of 6.24 years on unseen testing samples. To explore the age-related expression changes and evaluate the potential of age predictor to select therapeutic targets, authors compared a comprehensive set of ranking methods to identify protein-coding genes related to muscle aging.

While most of the progress has been made in the development of deep molecular aging clocks, the facial aging prediction is also a promising diagnostic tool. A research lab of the computer vision company Haut. AI proposed a deep photographic aging clock, PhotoAgeClock, developed using a set of over 8,000 anonymized high-resolution eye corner images [38]. Achieving a mean absolute error 2.3 years on the previously unseen by the model testing set, this age predictor is yet the most accurate. The proposed approach also was able to identify facial areas exposing age the most, with the skin around eye being the most age-relevant area.

Most of the practical results and most impressive achievements in deep learning are in imaging [2] and medical imaging is not an exception. Imaging-based human age predictors were developed for a variety of applications and as early as the estimation of gestational age [39].

Many deep aging clocks are being developed with the practical forensic applications in mind. Forensic age predictors were developed using femoral bone mineral density [40]. Age predictors utilizing deep learning were also developed for the estimation of age of otoliths of fish [41].

Magnetic Resonance Imaging (MRI) data is commonly used to train the deep neural networks to predict the age of the patient [42]. This technique may be used for early diagnosis and staging of a variety of neurological disorders. There are early signs that this approach may work in Multiple Sclerosis (MS) [43]. Most recently an attempt was made to correlate the predicted age by MRI and chronological age in people with depression [44]. While no meaningful correlation was found, other data types may be more applicable for this task.

Physical activity data can be used to predict the person’s age. Age predictors were developed using the data collected for people walking on a sensor floor [45].

### Applications of deep aging clocks in the pharmaceutical industry

Recent advances in artificial intelligence are rapidly propagating into the pharmaceutical drug discovery and drug development practices. The intersection of recent advances in AI and aging research yields many new tools and applications for the pharmaceutical industry to exploit—at every step of the research and development process, as well as in personalization, marketing, and real-world evidence. Deep aging and longevity clocks can be applied in many areas of pharmaceutical research and development starting from biological data quality control and management to age-personalized medicine, clinical trials enrollment analysis and marketing [28].

**Figure 3:**
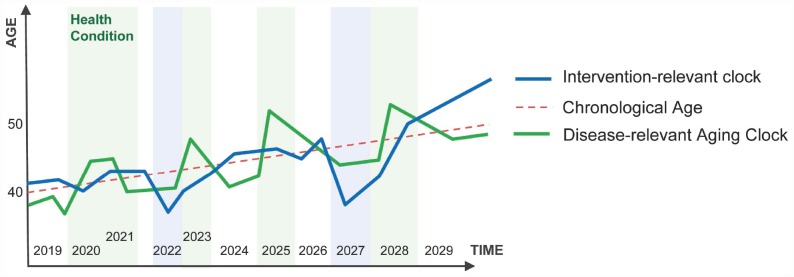
**Disease-relevant aging clocks and Intervention-relevant aging clocks.** The disease-relevant clock may indicate the presence or onset of a specific disease (e.g. the patient consistently "looks" older to the system then the chronological age). Intervention-relevant clocks may change in response to the intervention (e.g. the patient is consistently predicted younger than the chronological age in response to intervention).

One of the obvious applications of the deep aging clocks is evaluation of the sensitivity of a given data type to diseases and interventions. Some clocks trained on a specific data type may be relevant to the health status of the individual. For example, if the patients are consistently predicted older or younger than their chronological age during a specific disease using the hematological or transcriptomic aging clocks, these clocks are relevant to that disease and may be used for predicting the onset and stage of the disease. Other aging clocks may consistently predict the patients older or younger than their chronological age in response to a drug, gene or cell therapy, or another intervention. These clocks may be deemed to be intervention-relevant (Figure 3).

Some of the deep aging clocks may be very useful for predicting response or non-response to specific interventions. Many interventions in immuno-oncology rely on the state of patients’ immune system. The deep aging clocks may be used to track the immunosenescence levels, and identify and track new interventions designed to boost the immune system in the elderly. Biological age-personalization is another major potential application of the deep aging clock. The deep aging clocks can enable a way to track response rates. In clinical trials of meta-analysis that demonstrate patients predicted to be older than their chronological age respond better to an alternative dosage or vaccination protocol, necessary additional doses of the vaccine may be sold.

### Generation of synthetic data as a tool for target identification for aging

In addition to expanding the scope of aging clocks, neural networks can be used to generate synthetic data in large volumes. Generative Adversarial Networks (GANs), a popular new machine learning technique first introduced by Ian Goodfellow in 2014 [46] commonly used in drug discovery [3], enable the generation of biologically relevant synthetic data with specified conditions. For instance, breast cancer detection requires a large number of labeled mammograms to train convolutional neural network (CNN). Generally, this kind of requirement is infeasible for the collection of medicinal images especially for the mammographic tumor images. Hence, generation of synthetic data proposed a possibility to make the CNN classifier perform better [47]. Synthesizing new patient data using GANs trained on millions of samples, using only age as a generation condition, allows for massive anonymization of data while maintaining the most biologically relevant features.

As an ideal augmentation method, GANs give a comprehensive application in both medical and biological field. It also enables the identification of potential targets driving aging and disease-related processes [16]. Many diseases related to aging can be diagnosed using multiple omics data sets in different dimensions including transcriptomics data, which is usually used in targets identification process. Recently, a universal transcriptomic signature of age performed in Caenorhabditis elegans identified a handful of molecules which extend up to 30% of mean lifespan [48]. Combining the synthetic data of different age with other various data sets provides a novel approach to create an aging clock model with pathogenic or aging-related targets identified.

Multi-modal aging clocks obscure the difference between aging and disease status, essentially turning the many aging clocks into a marker of the health status of an individual. Since all living beings change over time, multi-modal aging clocks and clock ensembles trained on all accessible data types can act as a digital twin for a patient. This likeness can be moved forward and backward in time using GANs with multiple defined generation conditions including lifestyle choices and interventions. These clocks may also be embedded into field-trainable mobile devices that learn on the individual and help maintain an optimal biological age.

### Aging clocks for wellness programs

Discussions about the practicability to apply analysis of aging biomarkers [49] and data-driven behavior-tracking to health insurance risk control have been prevailing in the past decade. Meanwhile, regulations have left some room for preliminary innovations. The Affordable Care Act 2010 (ACA) and HIPAA generally interdict any means of discriminative pricing of group health plans to similarly situated individuals based on health factors such as health status, medical condition, medical history and etc. Nonetheless, HIPAA and ACA allow an exception for wellness programs under certain requirements [50].

Health insurers started to reward their members for better health habits. For instance, Oscar Health started to collect movement data through wearables, and offer $1 Amazon gift card every day to its member for hitting his or her step goal, up to $240 a year. However, the relationship between movement data and health has not been clinically established, and Oscar’s attempt to control or assess health risk with wearable data fell short of expectation [51].

A similar reward approach was proposed by VitalityHealth [52]. In addition to activity tracking, VitalityHealth also introduced a concept of Vitality Age providing rewards and benefits to individuals who chronologically are over 70, but predicted younger by simple survey-based test.

While different wellness programs are gaining popularity, identifying true healthy behavior with clinically established health risk mitigation or prevention remains a challenge. More fundamentally, insurers need a rigorous overall measure of human’s health risk and aging clocks can greatly benefit and strengthen current approaches.

### Deep aging and longevity clocks for preventative and therapeutic interventions in aging

**Figure 4:**
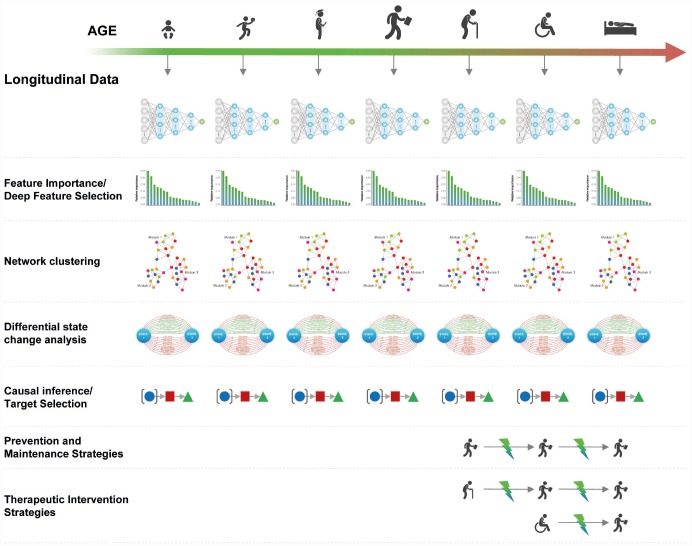
**Using age predictors within specified age groups to infer causality and identify therapeutic interventions**

Given a sufficient number of samples for training, DNN-based systems can be trained to predict age and health status on population-level multi-modal longitudinal data within specific age groups (e.g. decades 20–30, 30–40, 40–50). These DNNs can be used to identify the most important features, reconstruct the biological pathways, evaluate the state change in the pathways [7], and identify biological targets (Figure 4). This same process can be applied on the individual level by continuous retraining of the age and health status predictors on the individual's longitudinal data.

The causal targets and networks identified using this approach may be used to develop interventions for keeping the state of the network as close to the age group with optimal performance (20–30) as possible. These interventions may be used for prevention of the age-related pathological changes.

This approach of incremental age- and health-status prediction may be used to identify pathological changes transpiring during aging and identifying therapeutic interventions required to return the individual to the state resembling the optimal biological age.

## CONCLUSIONS

The deep biomarkers of aging and longevity have a broad range of applications in research and development, medical, insurance, and many other areas. Developing comprehensive granular multi-modal aging clocks will help get a better understanding of the aging processes, establish causal relationships, and identify preventative and therapeutic interventions. One of the many promising applications of the deep aging clocks built into the generative adversarial networks is generation of synthetic biological data with age as a generation condition. The deep aging clock research is expected to increase in popularity. Recent workshops at the National Institutes of Health (NIH) [53] and at the leading industry conferences [15] highlighted the need for development of the comprehensive deep age predictors. The authors expect this trend to continue with the inevitable commercialization in the insurance and consumer wellness industries.
